# QUALITY METRICS IN ALFS: FRAMEWORK, MEASURES, AND IMPLEMENTATION

**DOI:** 10.1093/geroni/igac059.1117

**Published:** 2022-12-20

**Authors:** Tetyana Shippee, Taylor Bucy, Odichinma Akosionu, Tricia Skarphol

**Affiliations:** University of Minnesota, Minneapolis, Minnesota, United States; University of Minnesota School of Public Health, Minneapolis, Minnesota, United States; University of Minnesota, Minneapolis, Minnesota, United States; University of Minnesota, Minneapolis, Minnesota, United States

## Abstract

Concerns have surfaced regarding the quality of assisted living (AL) with calls for quality metrics reporting as essential for consumer choice and organizational accountability. This study presents a framework for quality metrics in ALs using a) results from literature review and environmental scan of existing domains and indicators used to assess quality in AL and 2) a survey of results from MN stakeholders (n=822) on their priorities regarding these quality measures. Our findings showed that consumer-reported measures (resident quality of life and family satisfaction) were rated as top priority, followed by staff-based measures (e.g., job satisfaction, turnover). Other domains included residents’ safety, resident health outcomes, care services and integration, and the environment of the ALs. These results have implications for states looking to develop and implement quality measures in ALs, especially in the context of rising AL resident acuity and the continued trend away from institutional models of long-term care.

